# Validation and advantages of using novel RT-qPCR melting curve analysis assays for the identification of SARS-CoV-2 variants

**DOI:** 10.1038/s41598-022-17339-0

**Published:** 2022-07-29

**Authors:** Sebastian Juul, Malene Roed Spiegelhauer, Mette Neve Petersen, Katharina Kirkegaard Flugt, Nikolaj Vestergaard Hansen, Helene Larsen, Per Bo Jensen, Ulf Bech Christensen, Rasmus Koefoed Petersen, Lennart Friis-Hansen

**Affiliations:** 1 Research & Development, Pentabase A/S, Petersmindevej 1A, 5000 Odense C, Denmark; 2 Department of Clinical Biochemistry, Copenhagen University Hospital, Bispebjerg, Denmark; 3 Center for Diagnostics, DTU Health Technology, Technical University of Denmark, Kongens Lyngby, Denmark; 4 Institute of Clinical Medicine, University of Copenhagen, Copenhagen, Denmark

**Keywords:** Molecular biology, Infectious diseases

## Abstract

Reverse transcription quantitative PCR (RT-qPCR) assays are gold standard in diagnosing SARS-CoV-2 infection and play a major role in viral subtyping for rapid detection and monitoring of important mutations, containing the spread of new virus variants. We wanted to compare RT-qPCR melting curve analysis assays to Sanger Sequencing for detection of variants within the SARS-CoV-2 spike glycoprotein and examined their sensitivity and specificity. Samples positive for SARS-CoV-2 (n = 663 + 82) were subtyped using both Sanger sequencing and five RT-qPCR melting curve analysis assays specific for the mutations N501Y, P681H, E484K, K417N/T, and N439K. The results of the two methods were compared. The training cohort and the clinical validation cohort showed equally, or significantly better sensitivity of the assays compared to the Sanger sequencing. The agreement of the Sanger sequencing and the assays ranged from 92.6 to 100% for the training cohort and 99.4–100% for the clinical validation. The sensitivity, specificity, and turn-around time of the RT-qPCR melting curve analysis assays are well-suited for clinical monitoring of VOCs, making the assays an important tool in contact tracing and risk stratification. Furthermore, the assays were able to indicate the presence of new mutations in the complementary sequence to the mutation-specific probes.

## Introduction

Severe acute respiratory syndrome coronavirus 2 (SARS-CoV-2) is a novel betacoronavirus sharing sequence identity with SARS-CoV and MERS-CoV. It is an easily transmissible and pathogenic virus causing the coronavirus disease 2019 (COVID-19)^1^. The clinical spectrum of COVID-19 ranges from asymptomatic to viral pneumonia, that in some cases develops into acute respiratory failure with a high mortality rate^2^. SARS-CoV-2 was first described in Wuhan, China in late 2019, but has since spread and become a global pandemic^3,4^. The 30 kb SARS-CoV-2 genome encodes six open reading frames for structural proteins including the Nucleoprotein (N protein), Envelope (E protein) and Spike glycoprotein (S protein)^1^. The S protein consists of two functional subunits, S1 and S2. S1 is responsible for recognizing and binding to the host cell receptor, angiotensin-converting enzyme 2 (ACE2), while S2 mediates membrane fusion. The mutation rate for RNA viruses is high which allows for viral adaptation in regard to infectivity and immune escape, and is therefore correlated with virulence^5^.

## SARS-CoV-2 mutations and variants

Throughout the pandemic, the SARS-CoV-2 genome has gained mutations that increase the transmissibility and reduce the neutralizing effect of antibodies induced by vaccines or COVID-19. This has allowed the virus to spread even in populations that have already achieved herd immunity through infection or vaccinations^6^. Even though mutations can arise in all viral genes and cause amino acid alterations^7^, it is especially the amino acid changes in the receptor binding domain (RBD) of the S protein that have attracted great focus^8^ since these variations can increase the binding affinity to the ACE2 receptor^9^, thereby increasing viral transmission^10^. S protein variation also affects the binding of antibodies generated in response to infection with the SARS-CoV-2 strains and antibodies induced by vaccines based on the original S gene sequence affecting their ability to neutralize the virus^11^. The S gene variations give rise to SARS-CoV-2 variants of interest (VOI) and variants of concern (VOC) e.g. the distinct variants B.1.1.7 (Alpha), B.1.351 (Beta), P.1 (Gamma) and B.1.617.2 (Delta) that have increased infectivity and reduced neutralization by antibodies induced by SARS-CoV-2 vaccines^12,13^. This increased infectivity is in part due to specific amino acid changes such as K417N/T, N439K, E484K, N501Y and P681H caused by single nucleotide polymorphisms (SNPs) in the S-gene^14^. In order to contain, prevent or postpone the spread of new concerning SARS-CoV-2 variants by contact tracing, it is important that the turn-around time for test results is short, often 3–6 h and at maximum 24 h.

## Monitoring of VOC

The reverse transcription quantitative PCR assay (RT-qPCR) is the current gold standard molecular test for detecting the SARS-CoV-2 virus, and hundreds of different RT-qPCR assays have been designed and received Food and Drug Administration (FDA) Emergency Use Authorization (EUA) approval for COVID-19 diagnostics^15^. In the beginning of the pandemic, the focus was on detecting SARS-CoV-2 virus in samples using nucleic acid-based diagnostic assays that target the E, S, RNA-dependent RNA polymerase (RdRp), N, and open reading frame (ORF1ab) genes^16^. Initially, the genetic characterization was mainly done by genome sequencing and mainly to monitor the epidemiology of the outbreaks^17,18^. But as both the pandemic and the virus have evolved, the epidemiological need for characterization of SARS-CoV-2 variants arose for differentiated contact tracing. Rapid risk assessment and for guiding choice of monoclonal antibodies therapy^19,20^. Jørgensen et al.^21^ describes monitoring of mutations and VOC by Sanger sequencing of a specific fraction from the S gene requiring approximately 24 h with the use of an external firm for the sequencing (Eurofins, Cologne, Germany). However, sequencing is often too slow to be clinically useful in contact tracing aimed at containing the spread of the virus and risk assessment e.g., differentiated patient isolation regimes, as it requires specialized staff and equipment. In contrast, RT-qPCR based approaches satisfy both the clinical need for fast turn-around time, do not require specialized machinery in already SARS-CoV-2 established laboratories and can be performed by less specialized staff^17^,^22^. There are PCR based methods for performing SNP base typing of SARS-CoV-2 such as target failure PCR and allele specific primer/probe PCR^23–25^. The three methods cannot be produced with a single primer set and a probe complicating the production of new assays for upcoming variants of concern, resulting in an increased timeline before the new variant can be detected with PCR.

**Figure 1:**
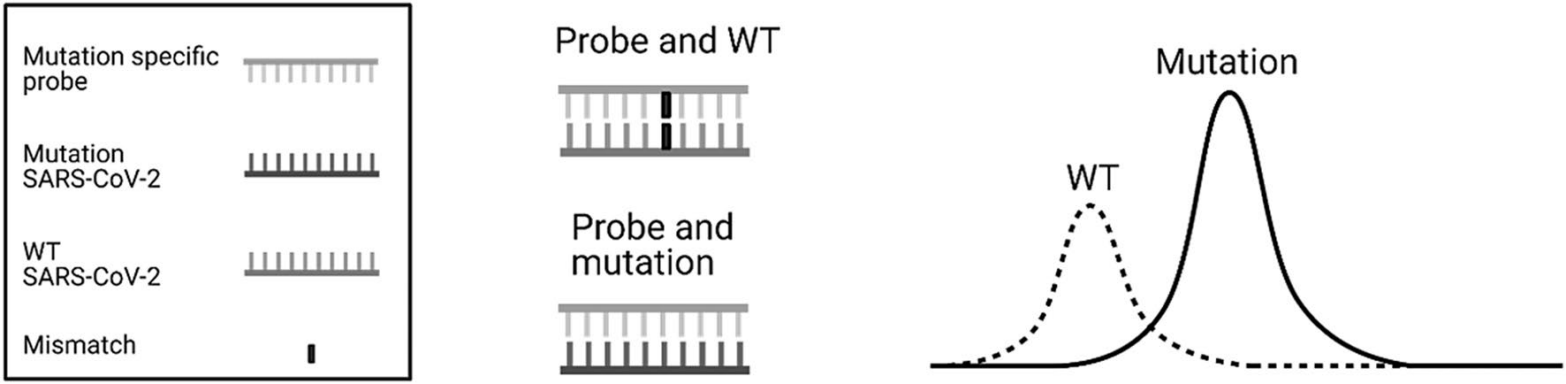
Created with BioRender.com The binding of the wild type and mutation sequence to the mutation specific probe. The probe has higher affinity for the mutation sequence and will result in a higher melting point temperature (Tm). The probe has a lowered affinity for the WT sequence due to the single nucleotide mismatch resulting in a decreased Tm.

To meet the clinical needs in Denmark, a rapid, simple, sensitive, and cost-effective RT-qPCR melting curve analysis assay was developed by PentaBase A/S (Odense, Denmark) for detection of SNPs in the SARS-CoV-2 genome. These assays are based on an EasyBeacon™^26^ probe. EasyBeacon™ probes are the PentaBase alternative to molecular beacon probes but without the addition of a self-complementary stem sequence. EasyBeacon™ probes are based on the Intercalating Nucleic Acid® (INA®) technology resulting in nuclease resistance and temperature independent quenching as well as increased signal to noise ratio compared to standard molecular beacons. The EasyBeacon™ is designed to recognize the specific mutation with 100% sequence identity, resulting in the highest possible affinity for the mutated SARS-CoV-2 strand. When the mutation-specific probe binds to the wild type (WT) SARS-CoV-2 sequence with lower affinity, the temperature needed to separate probe and strand, known as the melting temperature (Tm), is decreased as shown in Fig. 1. In this study, we wanted to evaluate PentaBase’s RT-qPCR melting curve analysis assays for the determination of the SNPs leading to K417N/T, N439K, E484K, N501Y and P681H mutations in the S gene of SARS-CoV-2 samples by comparing these results to the reference Sanger sequencing method.

## Methods

### Samples

Two datasets were used: From 2020-12-30 to 2021-04-17, 49,514 tests were performed at PentaBase A/S, Odense, Denmark, where 287 were positive for SARS-CoV-2. Of these positive samples, 82 were homogeneously selected as a training cohort for technical validation. From 2021-03-01 to 2021-05-24, 86,895 tests were performed at Bispebjerg Hospital, Copenhagen, Denmark. A total of 714 positive samples were detected, of which 693 were used for the clinical validation. The samples were collected as a mixture of oro- and nasopharyngeal swabs using Universal Transport Medium (UTM) and Viral Transport Medium (VTM) collection kits. All methods were carried out in accordance with the Danish National Board of Health’s guidelines and regulations. The study was approved as a quality assurance study by the institutional review board at Bispebjerg Hospital and since the study for both institutions does not include patient specific data it was exempted from obtaining informed consent from all subjects and/or their legal guardian(s).

### RNA extraction

200–300 µL of the collection media was transferred to the BasePurifier extraction system (PentaBase A/S, Odense, Denmark). In brief, this is a 4-step method utilizing a magnetic bead-based system for RNA and DNA purification. The steps include binding of the nucleic acids to the beads, 2 × wash and an elution resulting in co-purified RNA and DNA in 80 µL buffer (Xi’an Tianlong Science & Technology Co., Xi’an, China, Ltd. Viral DNA and RNA Extraction Kit Instruction Manual Version A/3 (2020)). 5 µL of the final eluate was used as template in each PCR reaction.

### SARS-CoV-2 RT-qPCR analysis

The RT-qPCR was performed at PentaBase using the CoviDetect™ Fast assay (PentaBase A/S, Odense, Denmark) on the BaseTyper (PentaBase A/S, Odense, Denmark) and the BaseTyper software version 1.0.208. The RT-qPCR program was: reverse transcription for 3 min at 52 °C, then a hot-start polymerase activation at 95 °C for 30 s, followed by 45 cycles of 90 °C for 1 s and 60 °C for 12 s.

At Bispebjerg Hospital, the RT-qPCR reactions were performed using the CoviDetect™ assay (PentaBase A/S, Odense, Denmark) on the CFX96 (Bio-Rad, Foster City, CA, USA) using the software Bio-Rad CFX Maestro (version 4.1.2433.1219). The RT-qPCR program was: Reverse transcription for 5 min at 52 °C, then a hot-start polymerase activation at 95 °C for 10 s, followed by 7 cycles of 95 °C for 5 s and 66 °C for 30 s, and finally 38 cycles of 95 °C for 5 s and 60 °C for 30 s.

### Detection of mutations by RT-qPCR melting curve analysis assays

The CoviDetect™ Variant simplex assays (PentaBase A/S, Odense, Denmark) are designed with a single primer set and an EasyBeacon™ probe^26^ (PentaBase A/S). The multiplex assays are a combination of two simplex assays containing two primer sets and two EasyBeacon™ probes^26^ (PentaBase A/S). The sequences of the primers and probes are listed in Table 1. 5 µL eluate from the BasePurifier was mixed with the RT-qPCR melting curve analysis assays containing 10 µL 2 × AmpliSmaRT One-Step RT-qPCR Master Mix (PentaBase A/S, Odense, Denmark) and 5 µL 4 × primer/probe mix. The RT-qPCR was performed using the following program: Reverse transcription 5 min at 52 °C, then a hot start polymerase activation at 95 °C for 30 s, followed by 45 cycles of 95 °C for 15 s and 60 °C for 45 s, followed by a continuous melting curve analysis: 95 °C for 1 min, 40 °C for 1 min, increasing the temperature up to 80 °C with 10 readings/°C for the BaseTyper and 2 readings/°C for the CFX96.

**Table 1:** Primer and probe sequences for the RT-qPCR melting curve analysis assays.

Mutation	Sequence (5’ to 3’)
**K417N/T**	
Forward primer	GATCTCTGCTTTACTAATGTCT
Reverse primer	AGCCTGTAAAATCATCTGG^a^
Probe	CAATATTTCCAGTTTGCCCTG^a^
**N439K**	
Forward primer	TTACAGGCTGCGTTATAGC
Reverse primer	CAAAAGGTTTGAGATTAGACTTCC
Probe	AATTCTAAAAATCTTGATTCTAAGG^a^
**E484K**	
Forward primer	CCTGTATAGATTGTTTAGGAAGTCTA
Reverse primer	CCATATGATTGTAAAGGAAAGT^a^
Probe	CACCTTGTAATGGTGTTAAAGG^a^
**N501Y**	
Forward primer	ACTTTCCTTTACAATCATATGG^a^
Reverse primer	CAGTTGCTGGTGCATGT G
Probe	GGTAACCAACACCATAAGTGG^a^
**P681H**	
Forward primer	GCAATGATGGATTGACTAGC^a^
Reverse primer	CCATTGGTGCAGGTATATGC
Probe	GCCCGCCGATGAGAATT^a^

The probes are dual probes with fluorophore in 5’ end and quencher in 3’ end.

^a^Modified with Intercalating Nucleic Acid^®^ (INA^®^) technology.

### Limit of detection (LOD)

The sensitivity of the RT-qPCR melting curve analysis assays was tested on synthetic RNA of the WT sequence. 20 replications of 50, 20, 10, 4, 2 and 0 copies/µL were tested in the evaluation of the LOD95%.

### Melting temperature

A theoretical study using synthesized complementary strands to the mutation specific probe was performed with both the mutations and the WT sequence to estimate the melting point for result interpretation.

### Sanger sequencing

The RT-qPCR prior to Sanger sequencing was set up in 20 µL reactions using 10 µL AmpliSmaRT One-Step RT-qPCR 2 × Master Mix (PentaBase A/S, Odense, Denmark), 5 µL 4 × primer mix and 5 µL eluate from the BasePurifier. The RT-qPCR reaction was performed using the following program: reverse transcription for 5 min at 52 °C, then hot start polymerase activation at 95 °C for 10 s, followed by 45 cycles of 95 °C for 5 s, 58 °C for 30 s, and 72 °C for 1 min, followed by 5 min at 72 °C. 1.5 µL of the unpurified PCR product along with 2 µL 10 µM sequencing primer diluted in 15 µL nuclease free water was shipped to Eurofins Genomics (Eurofins, Cologne, Germany) for Sanger sequencing using their Plate Seq Kit Mix. Two sequencing primers were designed to amplify from amino acid Asp17 and Thr385 in the S gene in the samples from Pentabase, covering the lower S protein and the RBD respectively. The sequencing primer described by Jørgensen et al.^21^ was used in the samples from Bispebjerg Hospital.

### Statistical analysis

Statistical analysis was performed using RStudio ggplot2, forcats, cowplot, dplyr, cutpointr, readxl and tidyr packages, version 1.4.1103.

### Performance criteria

For both the RT-qPCR melting curve analysis and the Sanger sequencing we define valid results as the tests in which the identification of examined variants was possible. A concordance of > 95% between Sanger sequencing and RT-qPCR melting curve analysis assays was defined as desirable and > 90% as acceptable.

## Results

### LOD

The assays ranged from a LOD of 20–100 copies for the simplex assays and 100–250 for the multiplex assays (Table 2). The LOD was not tested on mutation specific RNA, but due to the increased affinity for the mutated sequence, it was expected to be more sensitive.

**Table 2:** Limit of detection 95% of the assays.

Mutation	Limit of detection (copies)
K417N/T	100
N439K	20
E484K	20
N501Y	50
P681H	100
Multiplex N501Y	100
Multiplex P681H	250
Multiplex N439K	50

### Melting temperature

**Figure 2:**
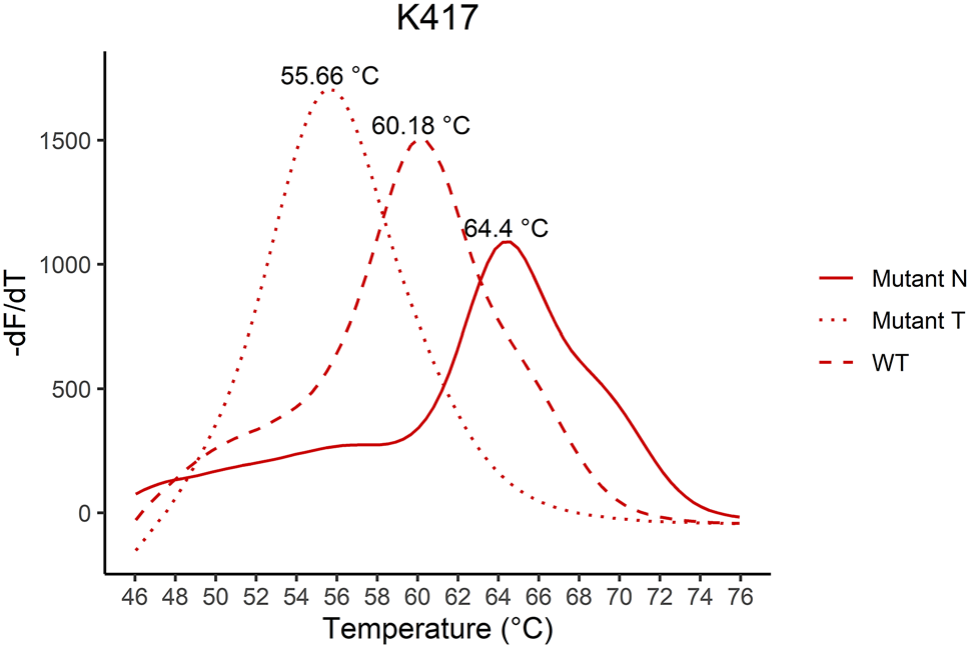
The melting curve analysis of the K417N mutation combined with a WT (K417), K417N and K417T complementary strand. The assay is specific for the K417N mutation resulting in the highest affinity for the sequence encoding Asparagine (N) in codon 417 and a Tm of 64.4 °C. The assay has the additional function as it can detect the K417T mutation at Tm 55.66 °C as well.

**Figure 3:**
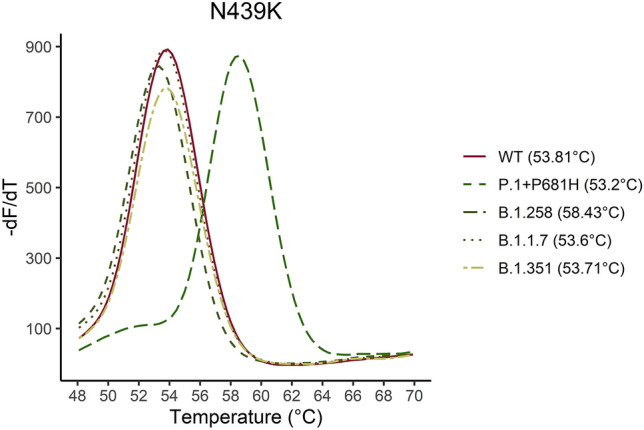
Five samples for the P.1 + P681H, B.1.351, B.1.1.7, B.1.258 and WT were analyzed. The melting curve of the five samples are illustrated for the N439K assay. As it is shown in the graph the N439K mutation can be distinguished by approximately 5 °C from the WT sequence.

**Figure 4:**
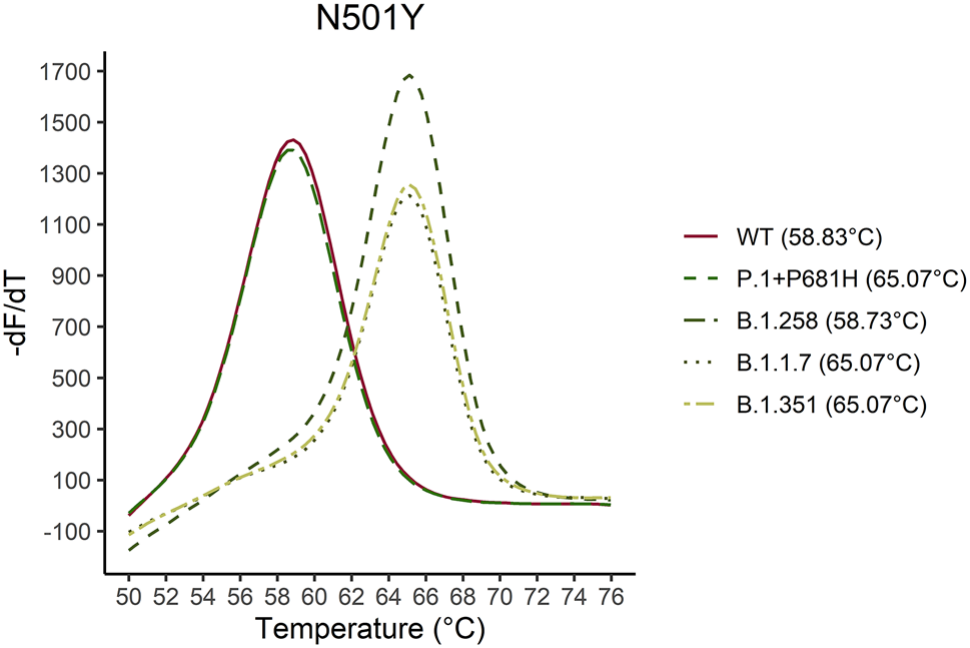
Five samples for the P.1 + P681H, B.1.351, B.1.1.7, B.1.258 and WT were analyzed. The melting curve of the five samples are illustrated for the N501Y assay. As it is shown in the graph the N501Y mutation can be distinguished by approximately 6 °C from the WT sequence.

**Figure 5:**
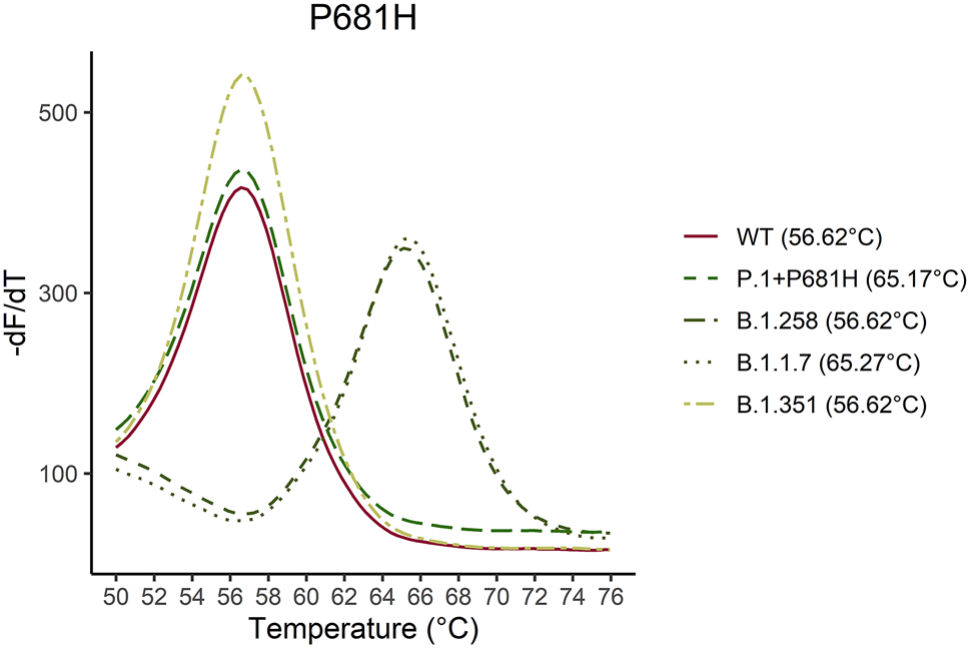
Five samples for the P.1 + P681H, B.1.351, B.1.1.7, B.1.258 and WT were analyzed. The melting curve of the five samples are illustrated for the P681H assay. As it is shown in the graph the P681H mutation can be distinguished by approximately 10 °C from the WT sequence.

**Figure 6:**
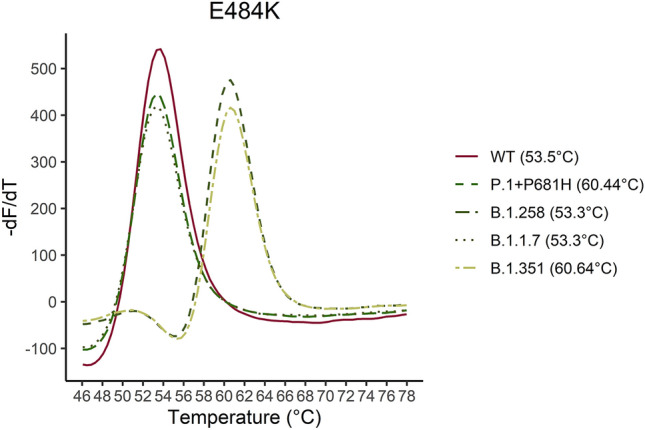
Five samples for the P.1 + P681H, B.1.351, B.1.1.7, B.1.258 and WT were analyzed. The melting curve of the five samples are illustrated for the E484K assay. As it is shown in the graph the E484K mutation can be distinguished by approximately 7 °C from the WT sequence.

**Figure 7:**
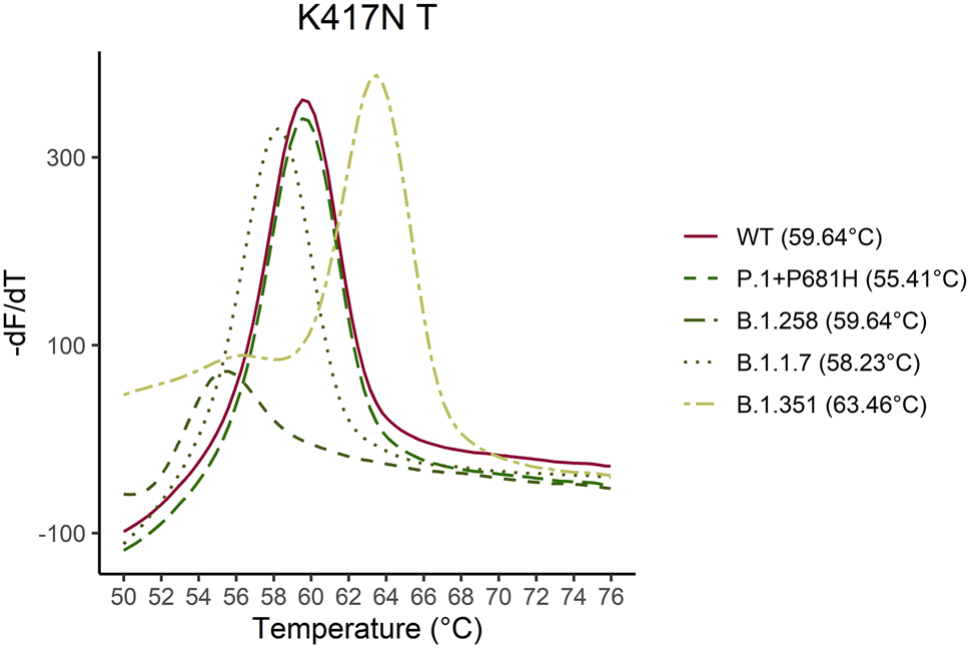
Five samples for the P.1 + P681H, B.1.351, B.1.1.7, B.1.258 and WT were analyzed. The melting curve of the five samples are illustrated for the K417N/T assay. As it is shown in the graph the K417N mutation can be distinguished by approximately 4 °C from the WT sequence and the K417T mutation can be distinguished 4 °C below the WT sequence.

Figure 2 shows melting curves with the estimated Tm for the K417, T417 and N417 sequences. The assay detects the K417T mutation at 53 °C as an additive function to the normal assays only detecting the WT amino acid and one amino acid substitution. The VOC and the B.1.258 were identified with the assays according to the affinity differences. In Fig. 3–7 samples harbouring WT, variants B.1.1.7, B.1.258, B.1.351, and P.1 respectively are illustrated (confirmed by Sanger sequencing), showing how the results are interpreted to differentiate the presence of a mutation. The melting temperatures for the different assays are listed in Table 3.

**Table 3:** Melting temperatures boundaries for mutations and WT sequences for the five assays.

N501Y		P681H		E484K	
WT	Mutation	WT	Mutation	WT	Mutation
56.0–60.5 °C	> 62.0 °C	55.0–58.5 °C	> 64.0 °C	52.0–55.0 °C	> 60.0 °C

N439K		K417N/T			
WT	Mutation	WT	Mutation N	Mutation T	
52.5–55.5 °C	> 58.0 °C	59.0–61.5 °C	> 63.5 °C	55.0–56.5 °C	

### Training cohort

The amount of SARS-CoV-2 in the 82 training cohort samples varied from almost no viral material (Ct values around 35) to a high amount of viral material (Ct values < 20).

The 82 samples were analyzed with the RT-qPCR melting curve analysis assays and Sanger sequencing.

The five assays K417N/T, N439K, N501Y, E484K, and P681H were used to detect the single nucleotide polymorphisms and to score the variants of concern B.1.1.7, B.1.351 and P.1 and B.1.258. The VOCs were scored with a minimum of mutations (Table 4). The assays showed a significantly better sensitivity compared to the Sanger sequencing (Table 5). The variant and RT-qPCR melting curve assay were similar in 92.6–100.0% (Table 6).

**Table 4:** A suggestion for the minimum of mutations required for identification of the listed SARS-CoV-2 substrains given the substrains present in Denmark from January to May 2021.

B.1.1.7	B.1.351	P.1	B.1.258
N501Y, P681H	N501Y, E484K, K417N	N501Y, E484K, K417T	N439K

**Table 5:** Comparison of the sensitivity for the RT-qPCR melting curve assay and Sanger sequencing for the training cohort.

All samples	PCR assay			Sanger sequencing			PCR vs. Sanger
	Conclusive	Inconclusive	%	Conclusive	Inconclusive	%	P-value
N501Y	80	2	97.6	56	26	68.3	1.814 × 10^–6^
E484K	78	4	95.1	55	27	67.1	1.146 × 10^–5^
K417N	76	6	92.7	55	27	67.1	9.801 × 10^–5^
P681H	78	4	95.1	47	35	57.3	3.746 × 10^–8^
N439K	81	1	98.7	56	26	68.3	4.338 × 10^–7^

Samples Ct > 31	PCR assay			Sanger sequencing			PCR vs. Sanger
	Conclusive	Inconclusive	%	Conclusive	Inconclusive	%	P-value
N501Y	27	2	93.1	12	17	41.3	8.937 × 10^–5^
E484K	25	4	86.2	12	17	41.3	0.001043
K417N	23	6	79.3	11	18	37.9	0.003361
P681H	25	4	86.2	9	20	33.3	6.359 × 10^–5^
N439K	28	1	96.6	11	18	37.9	7.593 × 10^–6^

The samples are divided into two groups: conclusive and inconclusive. Conclusive is when a result was available of the analysis and inconclusive was when a result was not available. The analysis is made for all the samples and the samples with a low amount of viral SARS-CoV-2 RNA (Ct > 31).

**Table 6:** Variant and mutation results from the RT-qPCR melting curve assays and the Sanger sequencing for the training cohort were compared how often they had similar results.

	Variant	N501Y	E484K	K417N/T	P681H	N439K
Similarity	95.9%	94.6%	92.6%	100.0%	100.0%	98.2%
n	49	56	54	55	47	56

Samples inconclusive for either the RT-qPCR assay or the Sanger sequencing are excluded.

Three of the samples that were not similar for the E484K assay had a mutation in another position below the probe, described as other mutation in the dataset. The sequencing data showed a mismatch in another nucleotide in the complementary sequence of the probe resulting in the lower melting temperature. The other samples that were not similar had low quality Sanger sequencing data.

### Clinical validation

The amount of SARS-CoV-2 in the samples used for clinical validation cohort varied from almost no viral material (Ct values around 35) to a high amount of viral material (Ct values < 20).

693 positive samples were analyzed with the RT-qPCR melting curve analysis assays and Sanger sequencing. RT-qPCR had 514 valid results, whereas the Sanger sequencing had 523. Only 466 of the samples had valid RT-qPCR and sequencing data (Table 7). The clinical validation cohort was analyzed with the four assays N439K, N501Y, E484K, and P681H. The K417N/T assay were not used at Bispebjerg Hospital.

**Table 7:** 714 positive samples were found at Bispebjerg hospital, 693 of the samples were analyzed using RT-qPCR melting curve assay and Sanger sequencing.

	Bispebjerg Hospital
n of samples	86,895
n of positive	714
n of positive with data	693
n of valid RT-qPCR typed (mutation assay)	514
n of valid Sanger sequencing typed	523
n of both valid RT-qPCR and sequence typed	466

466 of the samples could be analyzed with both methods.

The sensitivity of the two methods is compared in Table 8. The melting curve assay’s sensitivity was equally as good or significantly better than the Sanger sequencing. The low sensitivity on the assays P681H, N501Y and N439K was due to the use of the multiplex system of N501Y, P681H and N439K from samples analyzed at Bispebjerg Hospital. The LOD study (Table 2) reveals that the simplex assays are more sensitive than the multiplex assays. The sensitivity is lowered for the Bispebjerg Hospital samples (Table 8) relative to the training cohort (Table 5). 32.8% of the samples were inconclusive in either the mutation assay and/or Sanger sequencing. This might be due to the higher sensitivity of the CoviDetect™ and CoviDetect™ Fast assays with LOD of 20 and 5 copies respectively, making them able to detect smaller amounts of SARS-CoV-2 RNA. In Table 9 the samples lineage are determined for results with both valid PCR results and valid Sequencing data. The determination of the lineage from the sequencing results required that all the mutations were present. A suggestion for a minimum of mutations required to describe the variants using RT-qPCR data are explained in Table 4. The sequencing data and RT-qPCR data corresponded in 99.4% of the cases for the clinical validation cohort.

**Table 8:** Comparison of the sensitivity for the PCR assay and Sanger sequencing for the clinical validation cohort.

All samples	PCR assay			Sanger sequencing			PCR vs. Sanger
	Conclusive	Inconclusive	%	Conclusive	Inconclusive	%	
N501Y	547	146	78.9	557	136	80.3	0.5482
E484K	626	67	90.3	545	148	78.6	2.925 × 10^–9^
P681H	520	173	75.0	539	154	77.8	0.2548
N439K	253	112	71.8	533	160	76.9	1.827 × 10^–5^

Samples Ct > 31	PCR assay			Sanger sequencing			
	Conclusive	Inconclusive	%	Conclusive	Inconclusive	%	
N501Y	104	127	45.0	117	114	50.6	0.2637
E484K	170	61	73.6	109	122	47.2	1.147 × 10^–8^
P681H	77	154	32.2	106	125	45.9	0.007733
N439K	59	75	44.0	101	130	43.7	4.265 × 10^–7^

The samples are divided into two groups: conclusive and inconclusive. Conclusive is when a result was available of the analysis and inconclusive was when a result was not available. The analysis is made for all the samples and the samples with a low amount of viral SARS-CoV-2 RNA (Ct > 31).

**Table 9:** Variant and mutation results from the RT-qPCR melting curve assays and the Sanger sequencing for the clinical validation cohort were compared how often they had similar results.

	Variant	N501Y	P681H	N439K	E484K
Similarity	99.4%	100.0%	99.4%	100.0%	100.0%
n	466	493	475	209	524

Samples inconclusive for either the RT-qPCR assay or the Sanger sequencing are excluded.

## Discussion

We have validated five novel RT-qPCR melting curve analysis assays for rapid characterization of SNPs in the gene sequence for the SARS-CoV-2 spike protein and found their performance compared to Sanger sequencing satisfactory. Monitoring the spread of SARS-CoV-2 variants by genomic methods such as whole genome sequencing (WGS) or Sanger sequencing has become important tools for monitoring the SARS-CoV-2 pandemic. While these methods give substantial information about the presence of mutations, they are time-consuming and limit the potential for fast contact tracing. The use of RT-qPCR methods as an initial screening for mutations with subsequent confirmation by sequencing allows for a fast and specific detection of variants[^17,27^]. Characterization of SARS-CoV-2 has been used in Denmark to monitor the spread of multiple variants such as B1.1.7, B.1.519, B.1.525 and lately B.1.617.2 as directed by Statens Serum Institut^28^.

It was not possible to subtype all the positive samples. There could be several reasons for the difference between those detected and those subtyped. The assays CoviDetect™ and CoviDetect™ Fast are more sensitive with a LOD of 20 and 5 copies respectively and the melting analysis requires a higher amount of replicated genomic material for detection compared to conventional qPCR. This is demonstrated by the fact that samples presenting with high Ct values (low viral copy number) resulted in low quality of the Sanger sequencing data and were below the detection limit of the RT-qPCR melting curve analysis assays. While it is desirable to have the ability to subtype every sample containing virus, we experienced that 75–90% subtyping was enough to make a substantial impact on contact tracing and risk assessment^29,30^. The sensitivity of N439K, N501Y and P681H were lower in the Bispebjerg hospital cohort compared to PentaBase’s due to the use of the multiplex format at Bispebjerg. The use of the simplex assays would have increased the sensitivity of the analyses. The specificity of the multiplex and simplex assays was expected to be the same because the design of the primers and probes was unchanged (see Table 1). Despite the identical designs a little variation between the training and clinical validation cohorts’ specificity were observed but in both methods the specificity was found to be at least > 90% for the different assays. The difference was due to multiple samples with low sanger sequencing quality and a few samples with a lower melting temperature than the boundaries for wild type, detecting another mutation below the probe. As SARS-CoV-2 continues to spread throughout the globe and within communities, random nucleotide variations in both coding and non-coding areas of the viral genome are under continuous observation^31^. The assays are detecting genetic changes within the spike region which harbors higher variation compared to the relatively conserved N, E, RdRp and ORF1ab genes which most of the conventional RT-qPCR assays detects^32^. Normally, when designing RT-qPCR assays for the detection of SARS-CoV-2, primers should be placed in those regions with the least sequence variation and best conditions for PCR amplification^33^. When designing RT-qPCR assays for the identification of SARS-CoV-2 variants one is limited to designing the assays around the regions of interest, forcing the selection of less perfect regions for designing the primer sets^34^. This increases the risk of genetic variations within the primer sequences lowering the performance of the assay ultimately resulting in the analysis to fail. Bal, A. et al. describes the failure to detect the S protein gene in several Sars-CoV-2 samples by a triplex commercial kit^35^. The Ct-values for detection of the two other genes (N protein and ORF1ab) were low, indicating that sufficient viral material was present in the samples. However, the deletion of amino acids in the S gene was confirmed by WGS. The strains where the S protein gene could not be detected were found to contain the amino acid deletions H69del and V70del. Several other studies have reported detection failure in the presence of SNPs in target genes (E-gene detection failure at Cobas 6800 platform^36^, N- and E-gene mutations causing detection failure at Cepheid Xpert Xpress platform^37^).

In 466 samples (Table 7 714 positive samples were found at Bispebjerg hospital, 693 of the samples were analyzed using RT-qPCR melting curve assay and Sanger sequencing. 466 of the samples could be analyzed with both methods.), the RT-qPCR assays and the Sanger sequencing data matched, and the assays were found to be more or equally as sensitive as the Sanger sequencing while being less time consuming.

A few samples had genetic variation beside the desired mutation at the probe binding site, resulting in a decreased melting temperature compared to the WT. The samples with a genetic variation beside the desired mutation under the mutation specific probe were labeled as a mismatch in the RT-qPCR data even though it was detected with a lower melting temperature, as it was not introducing the indicated amino acid substitutions. The sanger sequencing analysis confirmed the suspected the genetic variation. The RT-qPCR assays had 99.4–100% similarity with the Sanger sequencing. In contrast to allele specific PCR assays, RT-qPCR melting curve analysis assays in several instances not only provide the ability to detect the targeted variation, but also allow for the detection of mutations in either the same codon or in neighboring codons^38^. In the data analysis, the P681H assay detected two P681R mutations. The change leading to the arginine amino acid (R) resulted in a shift in melting temperature in between the P681 and the H681 coding sequences. The R681 amino acid change was validated with a synthesized complementary strand and sequencing data. The assays’ ability to find and define new mutations is useful as the P681H assay might be used to detect the B.1.617.2 with the P681R amino acid change. The E484K assay had the capability to distinguish the E484Q mutation as well.

The emerging variants are characterized by numerous mutations, but the determination of a small number of mutations is often sufficient to describe the variants with high certainty. The similarity of the sequencing data and the assay regarding the variant was 99.4% thereby validating the minimum requirements described in Table 4. This results in a small number of assays needed for a total screening of a broad range of VOCs. PentaBase has recently developed an array of assays which has enabled us to track all of the major variants that have been circulating the Danish population e.g. B.1.1.7 (+ /−E484K), B.1.519, B.1.525 and B.1617.2 using assays that detect the mutations L452R, T478K, E484K, N501Y, P681H and Q677H. Banada et al. have also demonstrated the usefulness of RT-qPCR melting curve analysis assay for SARS-CoV-2 variant detection^39^.

Another PCR assay for identification of Sars-Cov-2 variants B.117, B.1351 and P1 has previously been described by Vogels et al.^40^. It is based on detection of the deletions Δ3675–3677 in the ORF1a gene present in all three variants, and Δ69–70 in the S gene of SARS-CoV-2 found in variant B.1.1.7. A study by Lind et al.^41^ compared the variant findings of this assay with whole genome sequencing. The assay was sufficient in identification of variants, but it could not distinguish between B.1351 and P.1 and would still rely on sequencing for an accurate identification. Anaclerio et al.^42^ describes another PCR based assay that can distinguish between the three VOC’s B.1.1.7, B.1.351 and P.1 by detecting the three mutations Δ69–70, E484K and N501Y simultaneity but it cannot distinguish between the B.1.351 and the P.1 variants. The assay used in this paper relies exclusively on RT-qPCR melting curve analysis assays of individual mutations and thus presents a large advantage in precise identification of each variant. The ease with which assays investigating new mutations can be developed also allows for a rapid introduction of new RT-qPCR melting curve analysis assays when new variants arise^17^. This is especially important in the period where new variants are introduced into a community as close monitoring of variant epidemiology, differentiated contact tracing and containment/isolation strategies mainly are of value as long as a given variant is restricted to limit to confined outbreaks and is not generally spread in the community^17^.

The results from some previous reports show that mutations and deletions may be present in the target sequence of the SARS-CoV-2 specific PCR assays resulting in failure of detection^35^, further supporting the need to screen for variants by RT-qPCR and subsequent confirmation by sequencing. As a result of the extensive sequencing of the SARS-CoV-2 genome, more than 2 million sequence entries have been submitted to GSAID^43^ and almost 1 million sequences have been submitted to NCBI^44^. In summary, in our daily clinical practice, we found that the combination of using RT-qPCR melting curve analysis assays for rapid specific variant analysis and sequencing (Sanger/whole genome sequencing) for variant surveillance very efficient.

A limitation of the assays is that they can first be designed once new variants have been identified by sequencing. Therefore, there is a one-two week delay due time needed for design and production before a new assay are ready for rapid detection of new variants.

Even though the virus strand identification table (Table 4) corresponded in 99.4% of the cases, new variants may cause a change to the table. For instance, the Omicron variants includes the N501Y and P681H mutations the same mutations as for Alpha in Table 4^45^. For distinguishing between them, new assays have been designed to overcome this problem. The CoviDetect variants are expanded with a lot of new assays for example L452R, T478K for the Delta variant^45^ and S371L, S373P, S375F for the Omicron variant and F486V for the most recent omicron BA.4/BA.5 variant^46^.

Overall, RT-qPCR melting curve analysis assays can be used as an effective tool in the detection of VOCs and mutations for rapid contact tracing. In contrast to sequencing, RT-qPCR melting curve analysis assays do not require specialized equipment or staff and can be implemented in most of the laboratories already screening for SARS-CoV-2 without investment in new machinery. The assays’ short turn-around time from collection to result time (< 3 h) can be decisive in ending chains of infection with more concerning variants compared to the much slower Sanger sequencing requiring 24 h.

In conclusion, we have demonstrated that RT-qPCR melting curve analysis assays provide a fast, flexible, reliable and cheap way of subtyping SARS-CoV-2 for fast virus strain identification and differentiated contact tracing facilitating containment of the spread of VOCs.

## Supplementary Information

Supplementary Information.

## Data Availability

The data that support the findings of this study are available in supplementary data.

## Code availability

The code that support the findings of this study are available upon request.
